# Crystal structure of 4-acetamido­benzoic acid monohydrate

**DOI:** 10.1107/S1600536814021886

**Published:** 2014-10-11

**Authors:** Wen-Juan Cai, Shao-Ming Chi, Jun-Feng Kou, Feng-Yi Liu

**Affiliations:** a College of Chemistry and Chemical Engineering, Yunnan Normal University, Kunming 650500, People’s Republic of China

**Keywords:** crystal structure, 4-acetamido­benzoic acid, hydrogen bonding, hydrated carb­oxy­lic acid

## Abstract

In the title compound, C_9_H_9_NO_3_·H_2_O, the plane of the acetamide group is oriented at 20.52 (8)° with respect to the benzene ring, whereas the plane of the carb­oxy­lic acid group is essentially coplanar with the benzene ring [maximum deviation = 0.033 (1) Å]. In the crystal, classical O—H⋯O and N—H⋯O hydrogen bonds and weak C—H⋯O hydrogen bonds link the organic mol­ecules and water mol­ecules of crystallization into a three-dimensional supra­molecular architecture.

## Related literature   

For applications of 4-acetamido­benzoic acid in coordination chemistry, see: Yin *et al.* (2011 ▶); Wang *et al.* (2009 ▶). For related structures, see: Kashino *et al.* (1986 ▶); Jedrzejas *et al.* (1995 ▶).
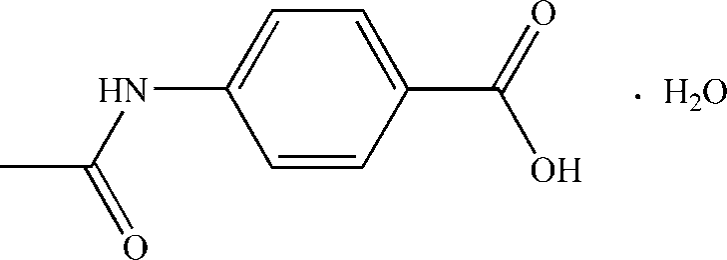


## Experimental   

### Crystal data  

C_9_H_9_NO_3_·H_2_O
*M* *_r_* = 197.19
Monoclinic, 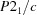

*a* = 6.6712 (13) Å
*b* = 28.870 (6) Å
*c* = 4.992 (1) Å
β = 100.01 (3)°
*V* = 946.8 (3) Å^3^
*Z* = 4
Mo *K*α radiation
μ = 0.11 mm^−1^
*T* = 293 K
0.31 × 0.28 × 0.26 mm

### Data collection  

Rigaku MM007-HF CCD (Saturn 724+) diffractometer
7525 measured reflections
1819 independent reflections
1448 reflections with *I* > 2σ(*I*)
*R*_int_ = 0.025

### Refinement  

*R*[*F* ^2^ > 2σ(*F* ^2^)] = 0.040
*wR*(*F* ^2^) = 0.116
*S* = 1.06
1819 reflections
140 parameters
1 restraint
H atoms treated by a mixture of independent and constrained refinement
Δρ_max_ = 0.16 e Å^−3^
Δρ_min_ = −0.21 e Å^−3^

### 

Data collection: *CrystalStructure* (Rigaku/MSC, 2006 ▶); cell refinement: *CrystalStructure*; data reduction: *CrystalStructure*; program(s) used to solve structure: *SHELXTL* (Sheldrick, 2008 ▶); program(s) used to refine structure: *SHELXTL*; molecular graphics: *DIAMOND* (Brandenburg, 1999 ▶); software used to prepare material for publication: *SHELXTL*.

## Supplementary Material

Crystal structure: contains datablock(s) I, new_global_publ_block. DOI: 10.1107/S1600536814021886/xu5824sup1.cif

Structure factors: contains datablock(s) I. DOI: 10.1107/S1600536814021886/xu5824Isup2.hkl

Supporting information file. DOI: 10.1107/S1600536814021886/xu5824Isup3.cml

**Fig. 1.**
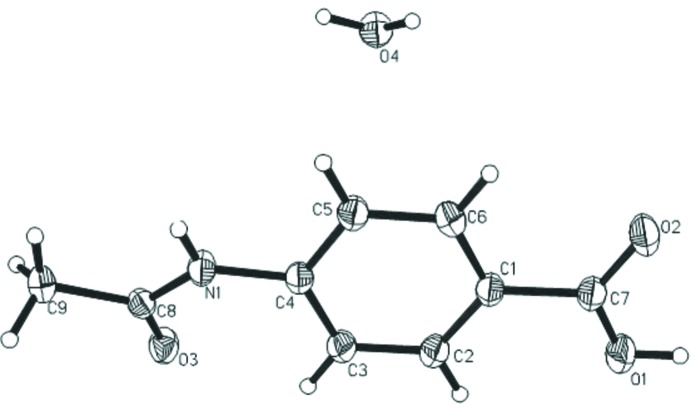
The molecular structure of the title compound, with atom labels and 30% probability displacement ellipsoids.

. DOI: 10.1107/S1600536814021886/xu5824fig1.tif
The mol­ecular structure of the title compound, with atom labels and 30% probability displacement ellipsoids.

**Fig. 2.**
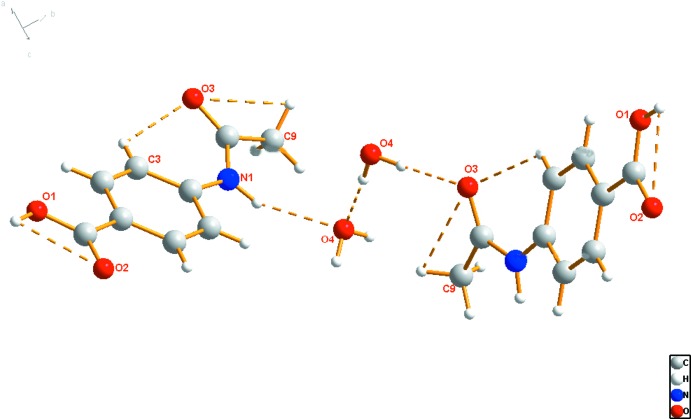
Hydrogen bonds are shown as brown dashed lines.

. DOI: 10.1107/S1600536814021886/xu5824fig2.tif
Hydrogen bonds are shown as brown dashed lines.

**Fig. 3.**
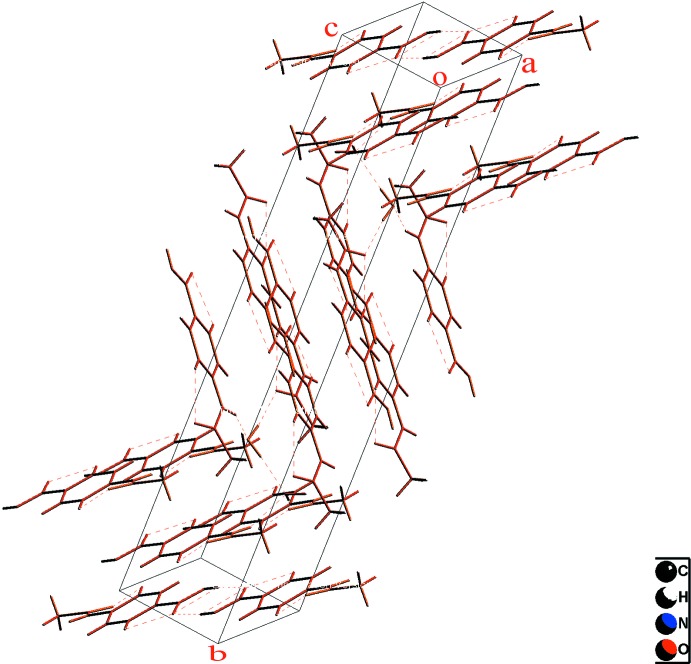
A view of the crystal packing.

. DOI: 10.1107/S1600536814021886/xu5824fig3.tif
A view of the crystal packing.

CCDC reference: 906509

Additional supporting information:  crystallographic information; 3D view; checkCIF report

## Figures and Tables

**Table 1:** Hydrogen-bond geometry (, )

*D*H*A*	*D*H	H*A*	*D* *A*	*D*H*A*
N1H4O4^i^	0.89(2)	2.17(2)	3.003(2)	155.4(17)
O1H1O2^ii^	0.88(2)	1.75(2)	2.6285(18)	174(2)
O4H2*W*O4^iii^	0.92(2)	1.98(2)	2.8920(14)	176(2)
O4H1*W*O3^iv^	0.83(3)	1.93(3)	2.7549(18)	173(2)
C9H9*C*O3^i^	0.96	2.57	3.484(2)	160

Symmetry codes: (i) 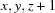; (ii) 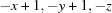; (iii) 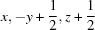; (iv) 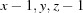.

## supplementary crystallographic information

### S1. Comment

In the past few years, 4-acetamidobenzoic acid has attracted increasing attention as a multifunctional ligand (Kashino *et al.*, 1986; Jedrzejas *et al.*, 1995). It shows potential diversified coordination and exhibits various coordination modes, such as monodentate, chelating, and multidentate bridging, *etc* in those reported complexes (Yin *et al.*, 2011; Wang *et al.*, 2009). Herein we report the synthesis and structure of the title compound.

The structure of the title compound is shown in Fig. 1, Fig. 2 and hydrogen bond geometry is given in Table 1. In the title compound, the acetamide moiety is oriented with respect to the benzene ring at 20.52 (8)° whereas the carboxyl group is essentially co-planar with the benzene ring [the maximum deviation = 0.033 (1) Å]. In the crystal, classic O—H···O, N—H···O hydrogen bonds and weak C—H···O hydrogen bond link organic molecules and crystalline water molecules into the three dimensional supramolecular architecture.

### S2. Experimental

4-Aminobenzoic acid (5 mmol, 686 mg) was added to 20 ml acetic anhydride. The mixture was then refluxed under argon for 8 h. The excess of acetic anhydride was then evaporated under reduced pressure. Water was then added to the resulting solid. The colorless block crystals were obtained after 48 h. Yield: 90% (based on 4-aminobenzoic acid).

### S3. Refinement

H atoms attached to N and O atoms were located in a difference Fourier map and refined with positional parameters, *U*_iso_(H) = 1.2*U*_eq_(N) and *U*_iso_(H) = 1.5*U*_eq_(O). H atoms attached to carbons were geometrically fixed and allowed to ride on the corresponding non-H atom with C—H = 0.96 Å, *U*_iso_(H) = 1.5*U*_eq_(C) for methyl H atoms and 1.2*U*_eq_(C) for other.

### Crystal data

**Table:**

C_9_H_9_NO_3_·H_2_O	*F*(000) = 416
*M**_r_* = 197.19	*D*_x_ = 1.383 Mg m^−^^3^
Monoclinic, *P*2_1_/*c*	Mo *K*α radiation, λ = 0.71073 Å
Hall symbol: -P 2ybc	Cell parameters from 8729 reflections
*a* = 6.6712 (13) Å	θ = 3.1–26.0°
*b* = 28.870 (6) Å	µ = 0.11 mm^−^^1^
*c* = 4.992 (1) Å	*T* = 293 K
β = 100.01 (3)°	Block, colorless
*V* = 946.8 (3) Å^3^	0.31 × 0.28 × 0.26 mm
*Z* = 4	

### Data collection

**Table:**

Rigaku MM007-HF CCD (Saturn 724+) diffractometer	1448 reflections with *I* > 2σ(*I*)
Radiation source: rotating anode	*R*_int_ = 0.025
Confocal monochromator	θ_max_ = 26.0°, θ_min_ = 3.1°
ω scans at fixed χ = 45°	*h* = −8→8
7525 measured reflections	*k* = −35→35
1819 independent reflections	*l* = −6→5

### Refinement

**Table:**

Refinement on *F*^2^	Primary atom site location: structure-invariant direct methods
Least-squares matrix: full	Secondary atom site location: difference Fourier map
*R*[*F*^2^ > 2σ(*F*^2^)] = 0.040	Hydrogen site location: inferred from neighbouring sites
*wR*(*F*^2^) = 0.116	H atoms treated by a mixture of independent and constrained refinement
*S* = 1.06	*w* = 1/[σ^2^(*F*_o_^2^) + (0.0607*P*)^2^ + 0.1929*P*] where *P* = (*F*_o_^2^ + 2*F*_c_^2^)/3
1819 reflections	(Δ/σ)_max_ < 0.001
140 parameters	Δρ_max_ = 0.16 e Å^−^^3^
1 restraint	Δρ_min_ = −0.21 e Å^−^^3^

### Special details

**Table:**

Geometry. All e.s.d.'s (except the e.s.d. in the dihedral angle between two l.s. planes) are estimated using the full covariance matrix. The cell e.s.d.'s are taken into account individually in the estimation of e.s.d.'s in distances, angles and torsion angles; correlations between e.s.d.'s in cell parameters are only used when they are defined by crystal symmetry. An approximate (isotropic) treatment of cell e.s.d.'s is used for estimating e.s.d.'s involving l.s. planes.
Refinement. Refinement of *F*^2^ against ALL reflections. The weighted *R*-factor *wR* and goodness of fit *S* are based on *F*^2^, conventional *R*-factors *R* are based on *F*, with *F* set to zero for negative *F*^2^. The threshold expression of *F*^2^ > σ(*F*^2^) is used only for calculating *R*-factors(gt) *etc*. and is not relevant to the choice of reflections for refinement. *R*-factors based on *F*^2^ are statistically about twice as large as those based on *F*, and *R*- factors based on ALL data will be even larger.

### Fractional atomic coordinates and isotropic or equivalent isotropic displacement parameters (Å^2^)

**Table:**

	*x*	*y*	*z*	*U*_iso_*/*U*_eq_	
C1	0.6802 (3)	0.43025 (5)	0.5354 (3)	0.0370 (4)	
C2	0.8849 (3)	0.42983 (5)	0.6534 (3)	0.0432 (4)	
H2	0.9751	0.4497	0.5886	0.052*	
C3	0.9563 (3)	0.40000 (5)	0.8671 (3)	0.0420 (4)	
H3	1.0932	0.4000	0.9459	0.050*	
C4	0.8203 (2)	0.37010 (5)	0.9622 (3)	0.0346 (4)	
C5	0.6157 (3)	0.37085 (6)	0.8459 (4)	0.0430 (4)	
H5	0.5249	0.3511	0.9107	0.052*	
C6	0.5461 (3)	0.40082 (6)	0.6342 (3)	0.0439 (4)	
H6	0.4086	0.4012	0.5578	0.053*	
C7	0.6019 (3)	0.46170 (5)	0.3049 (3)	0.0394 (4)	
C8	1.0650 (2)	0.32340 (5)	1.2903 (3)	0.0344 (3)	
C9	1.0717 (3)	0.29285 (6)	1.5361 (3)	0.0423 (4)	
H9A	1.1507	0.2657	1.5161	0.063*	
H9B	0.9358	0.2839	1.5528	0.063*	
H9C	1.1329	0.3095	1.6961	0.063*	
H1	0.684 (3)	0.5062 (7)	0.080 (4)	0.063*	
H4	0.775 (3)	0.3275 (7)	1.251 (4)	0.051*	
H1W	0.452 (4)	0.2928 (8)	0.293 (5)	0.063*	
H2W	0.545 (4)	0.2597 (7)	0.471 (5)	0.063*	
N1	0.8780 (2)	0.33893 (5)	1.1819 (3)	0.0386 (3)	
O1	0.7308 (2)	0.48727 (4)	0.2144 (3)	0.0554 (4)	
O2	0.4148 (2)	0.46159 (4)	0.2106 (3)	0.0546 (4)	
O3	1.21944 (18)	0.33300 (4)	1.1992 (2)	0.0475 (3)	
O4	0.5523 (2)	0.27530 (5)	0.3128 (3)	0.0486 (3)	

### Atomic displacement parameters (Å^2^)

**Table:**

	*U*^11^	*U*^22^	*U*^33^	*U*^12^	*U*^13^	*U*^23^
C1	0.0446 (9)	0.0327 (7)	0.0322 (8)	0.0049 (6)	0.0030 (7)	0.0027 (6)
C2	0.0428 (9)	0.0409 (8)	0.0444 (9)	−0.0030 (7)	0.0037 (7)	0.0100 (7)
C3	0.0355 (8)	0.0426 (8)	0.0452 (9)	−0.0011 (6)	−0.0006 (7)	0.0091 (7)
C4	0.0379 (8)	0.0341 (7)	0.0311 (7)	0.0040 (6)	0.0041 (6)	0.0028 (6)
C5	0.0369 (9)	0.0463 (9)	0.0445 (9)	−0.0028 (7)	0.0031 (7)	0.0120 (7)
C6	0.0384 (9)	0.0465 (9)	0.0428 (9)	0.0018 (7)	−0.0037 (7)	0.0064 (7)
C7	0.0490 (10)	0.0339 (7)	0.0338 (8)	0.0049 (7)	0.0033 (7)	0.0020 (6)
C8	0.0387 (8)	0.0331 (7)	0.0297 (7)	0.0021 (6)	0.0012 (6)	−0.0010 (5)
C9	0.0467 (10)	0.0463 (9)	0.0326 (8)	0.0083 (7)	0.0035 (7)	0.0071 (6)
N1	0.0358 (7)	0.0428 (7)	0.0369 (7)	0.0029 (6)	0.0056 (6)	0.0113 (5)
O1	0.0595 (9)	0.0540 (7)	0.0495 (7)	−0.0018 (6)	0.0003 (7)	0.0215 (6)
O2	0.0504 (8)	0.0573 (7)	0.0515 (7)	0.0061 (6)	−0.0043 (6)	0.0177 (6)
O3	0.0349 (6)	0.0563 (7)	0.0500 (7)	0.0022 (5)	0.0041 (5)	0.0156 (5)
O4	0.0409 (7)	0.0569 (8)	0.0496 (7)	0.0028 (5)	0.0122 (6)	0.0053 (6)

### Geometric parameters (Å, º)

**Table:**

C1—C6	1.386 (2)	C7—O2	1.255 (2)
C1—C2	1.390 (2)	C7—O1	1.274 (2)
C1—C7	1.487 (2)	C8—O3	1.228 (2)
C2—C3	1.389 (2)	C8—N1	1.347 (2)
C2—H2	0.9300	C8—C9	1.505 (2)
C3—C4	1.394 (2)	C9—H9A	0.9600
C3—H3	0.9300	C9—H9B	0.9600
C4—C5	1.388 (2)	C9—H9C	0.9600
C4—N1	1.4193 (19)	N1—H4	0.89 (2)
C5—C6	1.382 (2)	O1—H1	0.881 (16)
C5—H5	0.9300	O4—H1W	0.83 (3)
C6—H6	0.9300	O4—H2W	0.92 (2)
			
C6—C1—C2	119.44 (14)	O2—C7—O1	123.84 (14)
C6—C1—C7	119.22 (16)	O2—C7—C1	118.76 (15)
C2—C1—C7	121.34 (15)	O1—C7—C1	117.40 (16)
C3—C2—C1	120.74 (15)	O3—C8—N1	123.64 (14)
C3—C2—H2	119.6	O3—C8—C9	121.75 (15)
C1—C2—H2	119.6	N1—C8—C9	114.60 (14)
C2—C3—C4	119.27 (16)	C8—C9—H9A	109.5
C2—C3—H3	120.4	C8—C9—H9B	109.5
C4—C3—H3	120.4	H9A—C9—H9B	109.5
C5—C4—C3	119.93 (14)	C8—C9—H9C	109.5
C5—C4—N1	116.64 (14)	H9A—C9—H9C	109.5
C3—C4—N1	123.41 (15)	H9B—C9—H9C	109.5
C6—C5—C4	120.34 (15)	C8—N1—C4	128.98 (14)
C6—C5—H5	119.8	C8—N1—H4	116.6 (13)
C4—C5—H5	119.8	C4—N1—H4	114.4 (13)
C5—C6—C1	120.27 (16)	C7—O1—H1	117.5 (15)
C5—C6—H6	119.9	H1W—O4—H2W	104 (2)
C1—C6—H6	119.9		
			
C6—C1—C2—C3	0.6 (2)	C7—C1—C6—C5	179.16 (15)
C7—C1—C2—C3	−179.47 (15)	C6—C1—C7—O2	1.9 (2)
C1—C2—C3—C4	0.3 (3)	C2—C1—C7—O2	−178.08 (15)
C2—C3—C4—C5	−0.9 (2)	C6—C1—C7—O1	−177.98 (15)
C2—C3—C4—N1	−178.96 (15)	C2—C1—C7—O1	2.1 (2)
C3—C4—C5—C6	0.6 (3)	O3—C8—N1—C4	−3.9 (3)
N1—C4—C5—C6	178.79 (15)	C9—C8—N1—C4	176.06 (14)
C4—C5—C6—C1	0.3 (3)	C5—C4—N1—C8	163.45 (15)
C2—C1—C6—C5	−0.9 (2)	C3—C4—N1—C8	−18.4 (2)

### Hydrogen-bond geometry (Å, º)

**Table:**

*D*—H···*A*	*D*—H	H···*A*	*D*···*A*	*D*—H···*A*
N1—H4···O4^i^	0.89 (2)	2.17 (2)	3.003 (2)	155.4 (17)
O1—H1···O2^ii^	0.88 (2)	1.75 (2)	2.6285 (18)	174 (2)
O4—H2*W*···O4^iii^	0.92 (2)	1.98 (2)	2.8920 (14)	176 (2)
O4—H1*W*···O3^iv^	0.83 (3)	1.93 (3)	2.7549 (18)	173 (2)
C9—H9*C*···O3^i^	0.96	2.57	3.484 (2)	160

Symmetry codes: (i) *x*, *y*, *z*+1; (ii) −*x*+1, −*y*+1, −*z*; (iii) *x*, −*y*+1/2, *z*+1/2; (iv) *x*−1, *y*, *z*−1.

## References

[bb1] Brandenburg, K. (1999). *DIAMOND*. Crystal Impact GbR, Bonn, Germany.

[bb2] Jedrzejas, M. J., Luo, M., Singh, S., Brouillette, W. J. & Air, G. M. (1995). *Acta Cryst.* C**51**, 1910–1912. 10.1107/s0108270195003258 7576370

[bb3] Kashino, S., Matsushita, T., Iwamoto, T., Yamaguchi, K. & Haisa, M. (1986). *Acta Cryst.* C**42**, 457–462.

[bb4] Rigaku/MSC. (2006). *CrystalStructure*. Rigaku/MSC, The Woodlands, Texas, USA.

[bb5] Sheldrick, G. M. (2008). *Acta Cryst.* A**64**, 112–122. 10.1107/S0108767307043930 18156677

[bb6] Wang, Z. H., Fan, J. & Zhang, W. G. (2009). *Z. Anorg. Allg. Chem.* **635**, 2333–2339.

[bb7] Yin, X., Fan, J., Wang, Z.-H. & Zhang, W.-G. (2011). *Z. Anorg. Allg. Chem.* **637**, 773–777.

