# Supplementary figures and images for: Crystal structure of 4-acetamido­benzoic acid monohydrate
Source: Acta Crystallogr Sect E Struct Rep Online. 2014 Oct 11;70(Pt 11):o1154. doi: 10.1107/S1600536814021886 (PMC4257246; doi:10.1107/S1600536814021886)

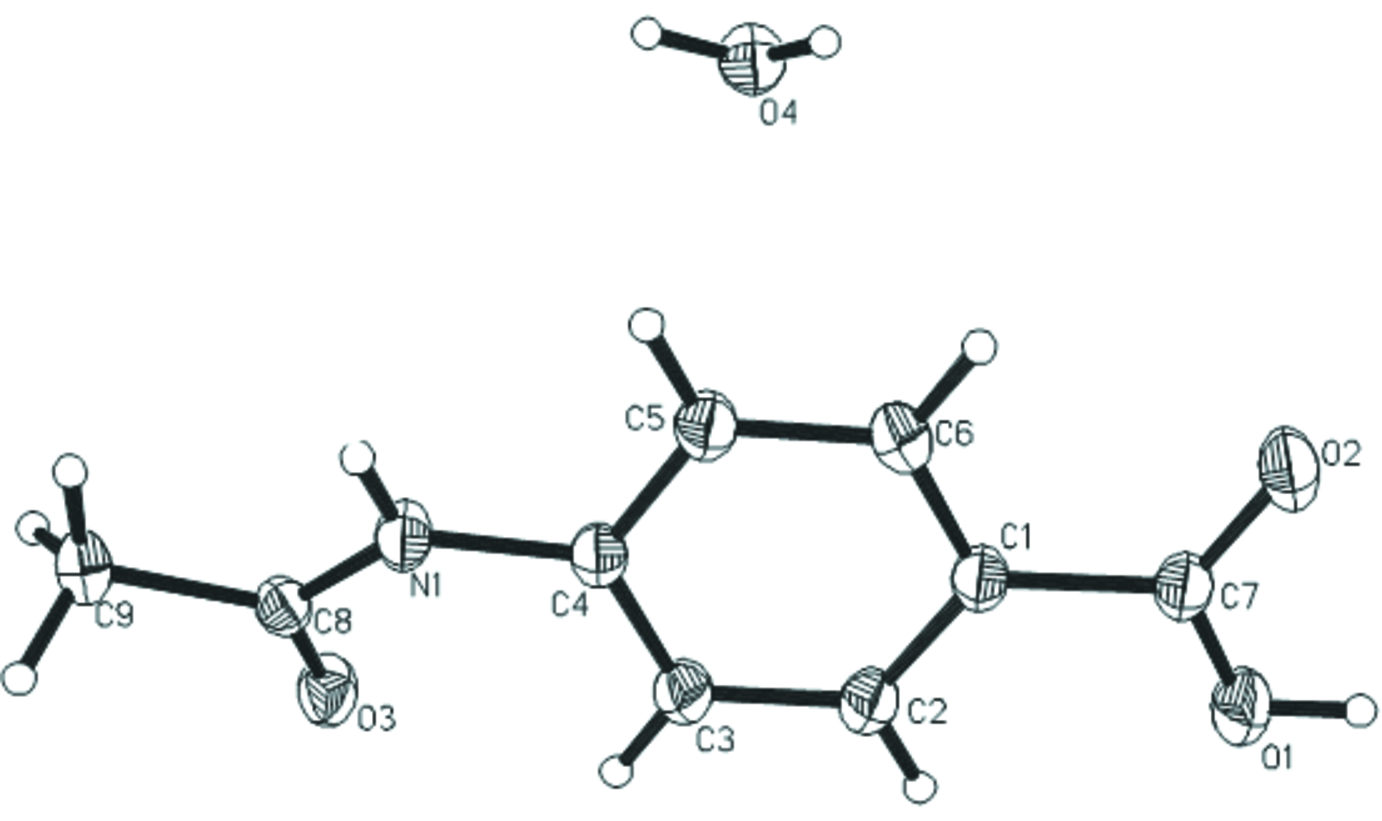

Supplement: Supplementary file 4 [file e-70-o1154-fig1.tif]

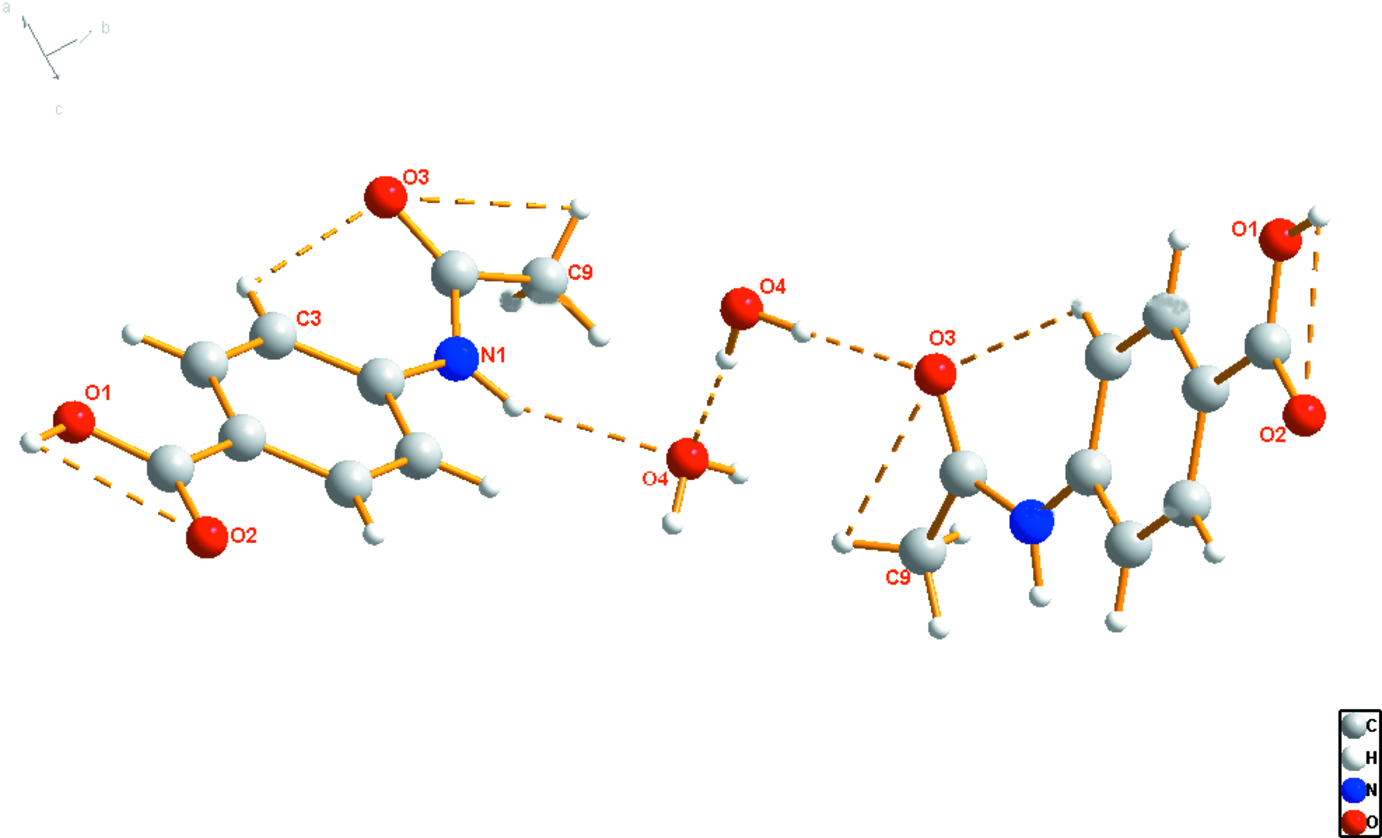

Supplement: Supplementary file 5 [file e-70-o1154-fig2.tif]

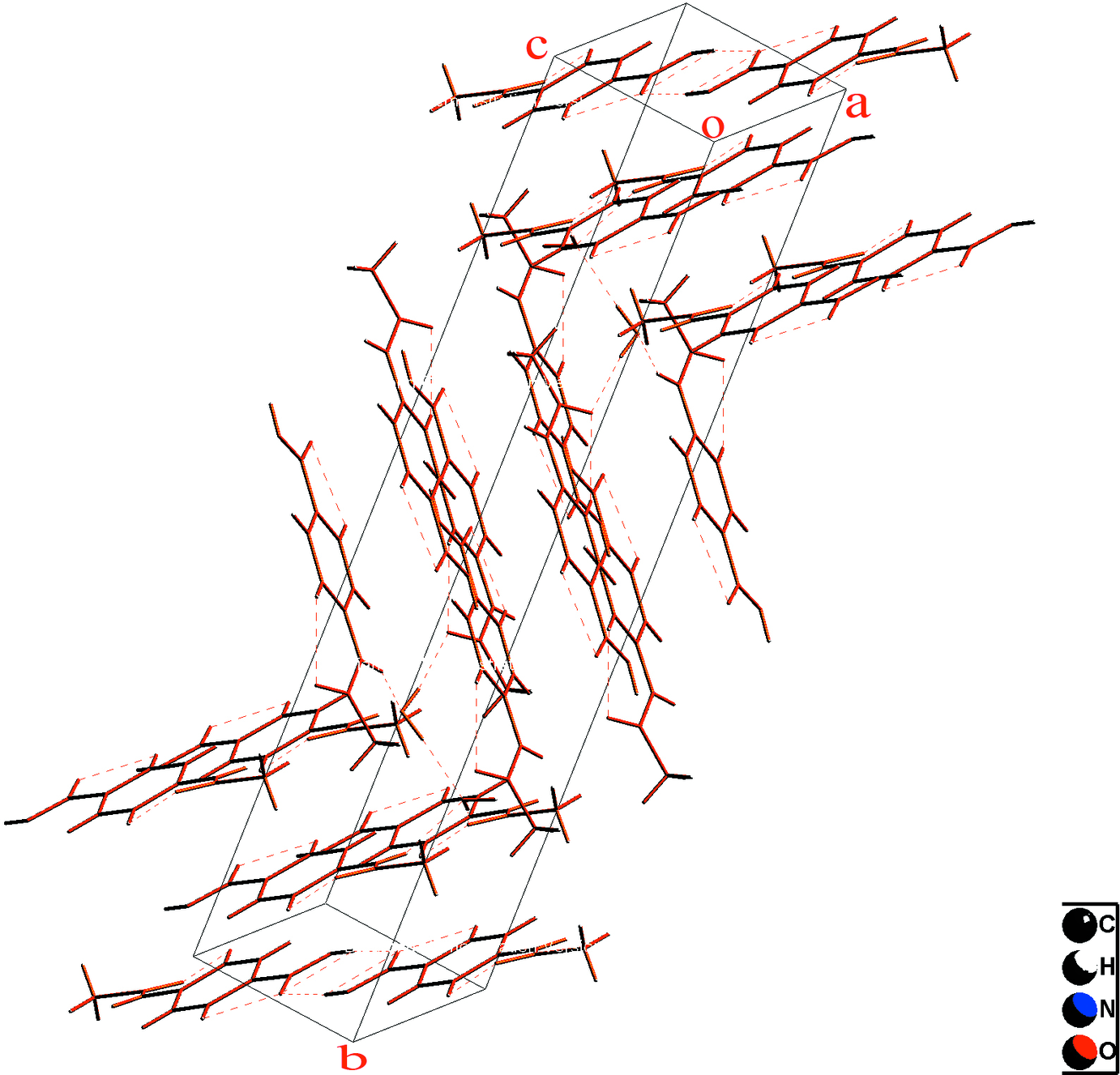

Supplement: Supplementary file 6 [file e-70-o1154-fig3.tif]
